# Spatial Pattern of Attacks of the Invasive Woodwasp *Sirex noctilio*, at Landscape and Stand Scales

**DOI:** 10.1371/journal.pone.0127099

**Published:** 2015-05-18

**Authors:** M. Victoria Lantschner, Juan C. Corley

**Affiliations:** Consejo Nacional de Investigaciones Científicas y Técnicas & Grupo de Ecología de Poblaciones de Insectos, INTA EEA Bariloche, Bariloche, Argentina; Instituto de Higiene e Medicina Tropical, PORTUGAL

## Abstract

Invasive insect pests are responsible for important damage to native and plantation forests, when population outbreaks occur. Understanding the spatial pattern of attacks by forest pest populations is essential to improve our understanding of insect population dynamics and for predicting attack risk by invasives or planning pest management strategies. The woodwasp *Sirex noctilio* is an invasive woodwasp that has become probably the most important pest of pine plantations in the Southern Hemisphere. Our aim was to study the spatial dynamics of *S*. *noctilio* populations in Southern Argentina. Specifically we describe: (1) the spatial patterns of *S*. *noctilio* outbreaks and their relation with environmental factors at a landscape scale; and (2) characterize the spatial pattern of attacked trees at the stand scale. We surveyed the spatial distribution of *S*. *noctilio* outbreaks in three pine plantation landscapes, and we assessed potential associations with topographic variables, habitat characteristics, and distance to other outbreaks. We also looked at the spatial distribution of attacked trees in 20 stands with different levels of infestation, and assessed the relationship of attacks with stand composition and management. We found that the spatial pattern of pine stands with *S*. *noctilio* outbreaks at the landscape scale is influenced mainly by the host species present, slope aspect, and distance to other outbreaks. At a stand scale, there is strong aggregation of attacked trees in stands with intermediate infestation levels, and the degree of attacks is influenced by host species and plantation management. We conclude that the pattern of *S*. *noctilio* damage at different spatial scales is influenced by a combination of both inherent population dynamics and the underlying patterns of environmental factors. Our results have important implications for the understanding and management of invasive insect outbreaks in forest systems.

## Introduction

Invasive forest insects can cause important damage to planted and natural forests, which can result in widespread tree mortality when pest population outbreaks occur [1–3]. Populations of outbreaking forest insects exhibit spatial patterns at multiple scales that can provide insights into why and how outbreaks develop. In turn, the spatial distribution of eruptive insect populations is affected by many factors that act at different scales, such as resource availability, habitat heterogeneity, climate, natural enemies, and insect dispersal behavior (see for example, [4–6]).

The spatial distribution of trees attacked by insects at a stand scale can be either randomly distributed or show some degree of clumping and this may vary with population size [7–10]. However at the landscape scale, insect populations that outbreak may show a spatial pattern that has an epicenter, where the outbreak begins and then spreads out through the forest or else, may occur as multiple simultaneous eruptions that tend to coalesce [3, 11, 12]. Variability in the spatial patterns observed and the variety of factors acting at different scales suggests that understanding the spatial pattern of attacks by forest pest populations is essential. This is not only because of its importance to improve our understanding of insect population dynamics in general but also because it is critical to predicting attack risk by invasives or planning pest management strategies.

*Sirex noctilio* is an invasive woodboring wasp that is native to Europe, northern Asia and Africa but that has established in several other regions of the world during the last century, such as Australasia, South America, South Africa, and North America [13–17]. This wasp has become one of the most important pests of softwood plantations in the Southern Hemisphere attracting much research aimed at controlling spread and preventing local outbreaks of established populations [18–23]. *Sirex noctilio* is a solitary hymenopteran insect that in association with a symbiotic fungus, *Amylostereum areolatum* and a phytotoxic venom, is the only siricid wasp capable of killing healthy host trees [13, 24, 25].

*Sirex noctilio* populations are known to remain at low densities in their native range and the insect is hence considered as a secondary pest there. However, in most of the invaded areas, this woodboring insect displays a pulse-like eruptive population behavior, which is when tree mortality becomes significant [13, 26]. Eruptive dynamics are characterized by lengthy periods during which the populations remain at relatively low densities and rapidly increase to outbreak levels in an unpredictable fashion [27]. Pulse-like outbreaks are rapid, they may last between 4 and 10 years, and are usually terminated by natural enemies, resource defenses or depletion [27].

The distribution and magnitude of *S*. *noctilio* outbreaks can be related to resource availability as well as to woodwasp life history traits [13, 28]. In this sense, Madden [13] proposed the “intermittent drought hypothesis” to explain the occurrence of *S*. *noctilio* outbreaks, according to which, discontinuous drought levels occurring during the woodwasp emergence season increase tree attractiveness and susceptibility to *S*. *noctilio* attacks. In contrast, Corley, Villacide and Bruzzone [7] suggest that limited dispersal behavior could lead to increased wood wasp performance through the concentration of attacks on trees, pontentially leading to local outbreaks (see also [28]).

While in general it is believed that any individual tree stressing factors may lead to enhanced susceptibility to woodwasp attacks, how these relate to outbreak magnitude and spatial population behavior is affected by scale. For instance, at a regional scale, extreme climatic events, like area-wide droughts may increase infestation probability by affecting tree vigor and susceptibility to insect attacks throughout a region [13]. At a finer scale, in turn, such as landscapes (defined as a spatially heterogeneous area of around 100 km^2^), topographic variables like elevation, slope, and slope aspect may also drive tree eco-physiological processes that may influence tree vulnerability. The degree of gross host availability (i.e., the amount of host species forest cover in an area) might also influence infestation occurrence. Finally, some stand-level factors, such as forest management (e.g. pruning, thinning) may determine the probability of trees to resist insect attacks [29, 30].

Although intensive research efforts have been carried out during the last decades toward understanding the biology, behavior, and control methods of *S*. *noctilio* (reviewed in [31]), there is still little information on the spatial distribution pattern of woodwasp attacks and its relationship with environmental factors at landscape or stand scale (but see [7][7]). By studying the outbreaks of current *S*. *noctilio* populations in NW Patagonia, our aims were: (1) to determine the spatial pattern of outbreaks and their relation with environmental factors at a landscape scale; and (2) to characterize the spatial pattern of trees attacked by *S*. *noctilio* at a stand scale. This information may contribute to a better understanding of the factors influencing *S*. *noctilio* outbreaks in general and also aid managers in predicting future infestations at both the stand and landscape scales.

## Methods

### Study area

The study was carried out in NW Patagonia (from 39° 15’ S to 43° 00’ S; and from 71° 00 W to 71° 34’ W). The climate of this area is temperate, dominated by a marked west-to-east decrease in precipitation. The study region is located in the forest/steppe transition area, where native vegetation ranges from forests of *Austrocedrus chilensis* and *Nothofagus antarctica* in the west, to a steppe dominated by bunchgrasses and low shrubs in the east. Mean annual rainfall ranges between 700 and 1,200 mm/year [32]. Pine plantations in the study region comprise mainly of three species: ponderosa pine (*Pinus ponderosa*), lodgepole pine (*Pinus contorta*), and Monterey pine (*Pinus radiata*).

### Spatial distribution of outbreaks at the landscape scale

**Fig 1:**
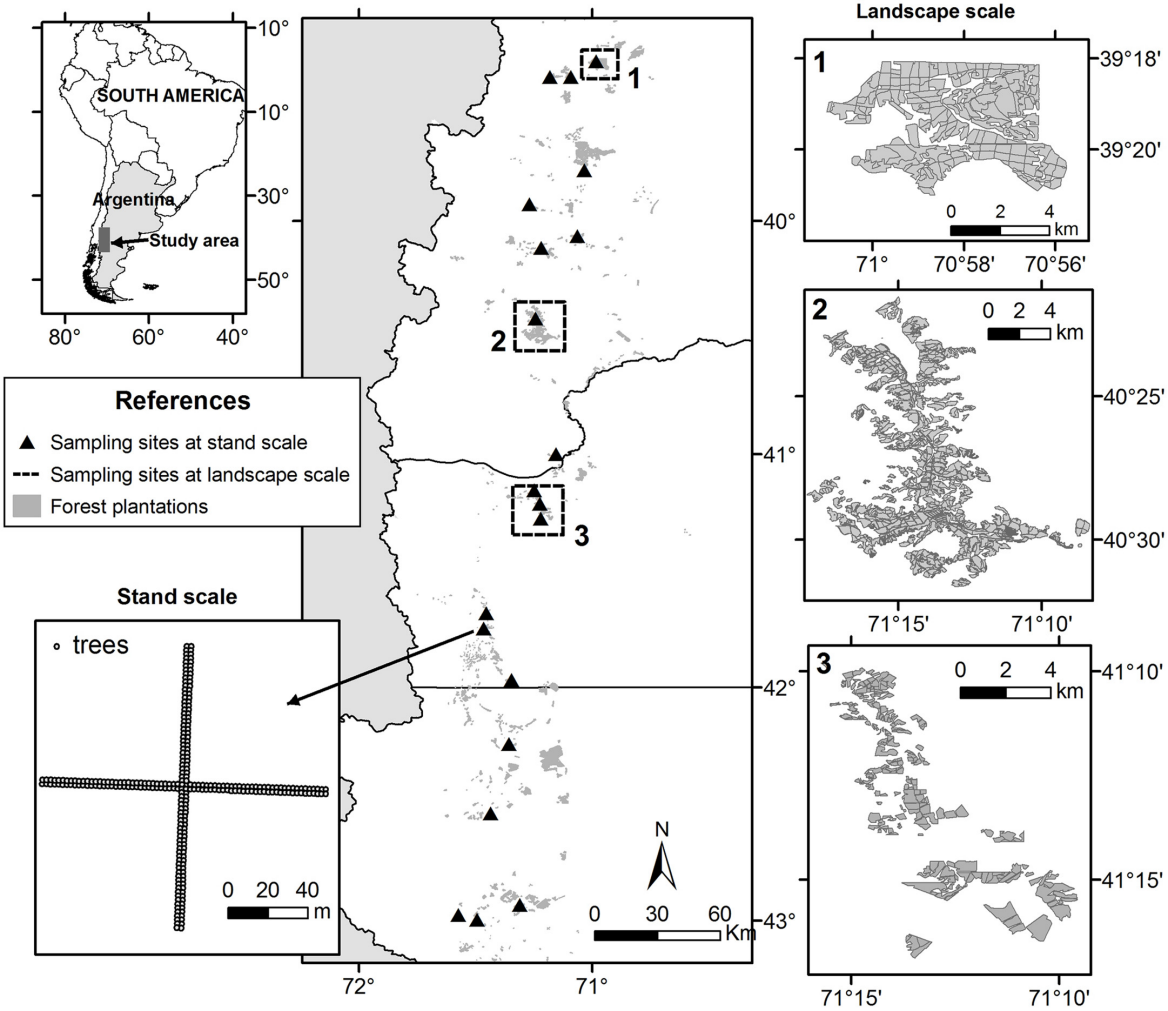
Location of the study area in Patagonia, showing sampling sites at landscape and stand scale.

We studied the distribution of *S*. *noctilio* outbreaks, in three of the largest pine plantation landscapes of Patagonia: *Arroyo del Medio* (41°10'- 41°16'S; 71°10'- 71°16'W), *Meliquina* (40°22'- 40°30'S; 71°09'- 71°17'W) y *Abra Ancha* (39°18'- 39°21'S; 70°55'- 71°00'W) (Fig 1). The study was carried out on private lands, with permission of the owners. At each of these afforested landscapes, we developed maps of *S*. *noctilio* outbreak distribution. We conducted extensive visual surveys of *S*. *noctilio* damage at each landscape, with vehicles and on foot, within each stand-defined as woodlands with species and ages susceptible to be attacked by *S*. *noctilio* of surfaces from 5 to 20 ha-. In each stand we identified the occurrence of current and recent outbreaks. We defined outbreaks as stands with more than 5% of trees attacked by *S*. *noctilio* [22]. Observations were made from hills and/or places with panoramic view of each stand, near enough to detect levels of individual dead and dying co-dominant/ dominant trees below 5%. We identified as attacked trees those with obvious browning of the needle, or foliar senescence. In Patagonia, *S*. *noctilio* is to our knowledge the only insect species known to kill healthy pines, and is the only pest that develops outbreaks in pine plantations causing extensive tree mortality. Thus, by observing patches of trees with typical symptoms of the woodwasp attack, we confidently assumed that it represented a *S*. *noctilio* outbreak (see also below).

Detailed ground surveys were conducted in 20 stands inside the surveyed areas to confirm the presence of *S*. *noctilio*, and to calibrate the remote visual detection of outbreaks. We randomly selected 10 stands previously classified as outbreaks and 10 stands previously classified as not-outbreaks. In each stand we randomly established 7 transects, maximizing the coverage of the area. At each transect we walked along a plantation line, examining 70 trees and recording the number of attacked trees (trees with emergence holes or resin droplets). Surveys were performed during the months of January to April 2013.

We considered a set of 12 environmental variables as potential predictors of variation in *S*. *noctilio* outbreak occurrence. Variables examined included 8 that were obtained for all the surveyed stands: presence of *P*. *ponderosa* or *P*. *contorta* in the affected stand, proportion of *P*. *ponderosa* or *P*. *contorta* in a 1km radius neighborhood, altitude, slope, aspect, and distance to the nearest outbreak.

We used 1:500,000 resolution maps of plantation forests in the study area [33, 34] to define the location and planted species in each stand. Landscapes around each stand were described using a circular area of radius = 1km. We use this size of buffer to evaluate *S*. *noctilio* perception of landscape features, based on prior knowledge. Recent work has shown that the dispersal distances of adult females—using intercept baited traps- into a non-pine habitat, does not exceed 1,000 m from the plantation edge (Villacide unpublished data). We calculated the proportion of *P*. *ponderosa* and *P*. *contorta* surface inside this buffer area.

We used 30 m resolution ASTER digital elevation models of the study area [35] to estimate the topographic variables (altitude, slope angle, aspect), using ArcGIS 9.2 software (ESRI, Redlands, California, USA). To assign one value of each variable for each stand, we used the midpoint of each stand.

We performed Moran’s I autocorrelation test to assess the existence of spatial autocorrelation of attacked stands along each sampled basin. The univariate global Moran’s I, measures the linear association of a variable with itself in space and was used to test the strength of global spatial autocorrelation of *S*. *noctilio* attacked stands in each region. Moran’s I ranges between −1 and +1. A positive Moran’s I, indicates spatial clustering of either high or low values, whereas a Moran’s I near zero implies no spatial autocorrelation, or spatial randomness.

We used multiple logistic regressions to assess how *S*. *noctilio* outbreaks are related to environmental variables at the landscape level. We used presence/ absence of outbreaks as the dependent variable and included environmental variables as independent variables. Prior to analysis, aspect values were transformed from circular variables to a measure relevant to vegetation as: aspect = cosine (45° —azimuth degrees) + 1 [36]. Values range from 0 on south-western slopes commonly exposed to the sun to 2 on the least exposed north-eastern slopes. To understand the correlational structure in our data, and to pre-select variables for model comparison, we performed Spearman’s correlations in a pairwise fashion between all predictor variables and with estimated spread rate. Variables that showed at least moderate correlation with spread (p>0.15) were selected. To avoid multicollinearity, we performed Spearman correlations between pairs of predictor variables, setting a limit of r_ρ_< 0.5 for keeping two correlated variables. In case of two variables been highly correlated, we excluded the variable less correlated with presence of *S*. *noctilio* outbreaks. All possible combinations of selected predictor variables were modeled, using logistic regression. The models were compared using Akaike’s Information Criterion for small sample sizes (AICc). Predictor variable pre-selection was necessary to allow us to employ an all possible regressions approach due to small sample size.

We calculated Ivlev’s electivity index (E_i_, [37][37]), to assess which host species were selected (0 < E_i_ ≤ 1) and which were avoided (-1 ≤ E_i_ < 0) by *S*. *noctilio* eruptive populations, at a landscape scale. Ivlevs electivity index (E_i_) was calculated as: E_i_ = (o_i_—l_i_)/(o_i_ + l_i_), where oi is the proportion of stands of host species i with *S*. *noctilio* outbreaks, and li is the proportion of stands of host species i in each studied landscape.

To assess the niche breadth of *S*. *noctilio* eruptive populations in terms of host use, we calculated the proportional similarity index (PS) or the Czekanowsky index [38]: PS = 1–0.5 Σ | pi - qi |, where pi is the proportion of stands composed by species i with occurrence of *S*. *noctilio* outbreaks; and q_i_ is the proportion of stands of species i, available to the population in each of the studied landscapes. The values for PS range from 1 for the broadest possible niche (a population uses resources in proportion to their availability) to 0 for the narrowest possible niche (a population is specialized exclusively on the rarest resource).

### Spatial distribution of attacked trees at stand scale

We randomly selected 20 sites distributed among pine plantations of NW Patagonia (Fig 1). At each site, we selected one stand attacked by *S*. *noctilio*, with the highest attack level possible to find, based on the observation of external symptoms (browning or dead trees). Sampling sites were separated by a minimum of 8 km from one another to ensure spatial independence among *S*. *noctilio* populations.

At each sampling site we established a point in the middle of the stand, and from this point we established four transects, one in each cardinal direction. Each transect consisted of two parallel tree lines, totaling 70 trees (280 trees per site, Fig 1). We identified and recorded the number of attacked trees (trees with emergence holes or resin droplets), specifying the species and relative position within the transect for each attacked tree. Surveys were performed during the months of January to April 2014.

We performed the non parametric Kruskal-Wallis test to check for differences in the proportion of attacked trees between tree species, followed by *post-hoc* multiple comparisons. We performed a non-parametric Mann-Whitney test, to assess if there were differences in the proportion of attacked trees between stands with different management practices (thinned vs. unthinned, and pruned vs. not pruned).

We used Moran’s I to measure spatial autocorrelation and to identify spatial clustering in the attacked trees along transects, at stand scale. We also performed a quadratic regression analysis of the relationship between Moran’s Index and % of *S*. *noctilio* attacked trees at each site, to assess how the spatial aggregation the attacked trees varied with the total percentage of attacked trees in the stand.

## Results

### Spatial distribution at the landscape scale

Overall, we sampled and mapped a total of 7350 ha of pine plantations, including 741 stands: 370 in *Meliquina* (3,642 ha), 205 in *Arroyo del Medio* (1,685 ha), and 161 in *Abra Ancha* (2,023 ha). Seventy-five percent of the sampled stands were composed of pure *Pinus ponderosa*, 12% were mixed stands of *P*. *ponderosa* and *P*. *contorta*, 8% were of pure *P*. *contorta*, and 4% mixed stands of several other species (*P*. *jeffreyi*, *P*. *radiata*, *Pseudotsuga menziesii*).

We identified the presence of *S*. *noctilio* outbreaks in 64 of the sampled stands. From the ground transects we confirmed that in all cases we correctly identified the presence of *S*. *noctilio* attacks from outside of the stand. Additionally, in 90% of the cases, we correctly identified the presence or absence of an outbreak as defined by Villacide and Corley [22].

**Fig 2:**
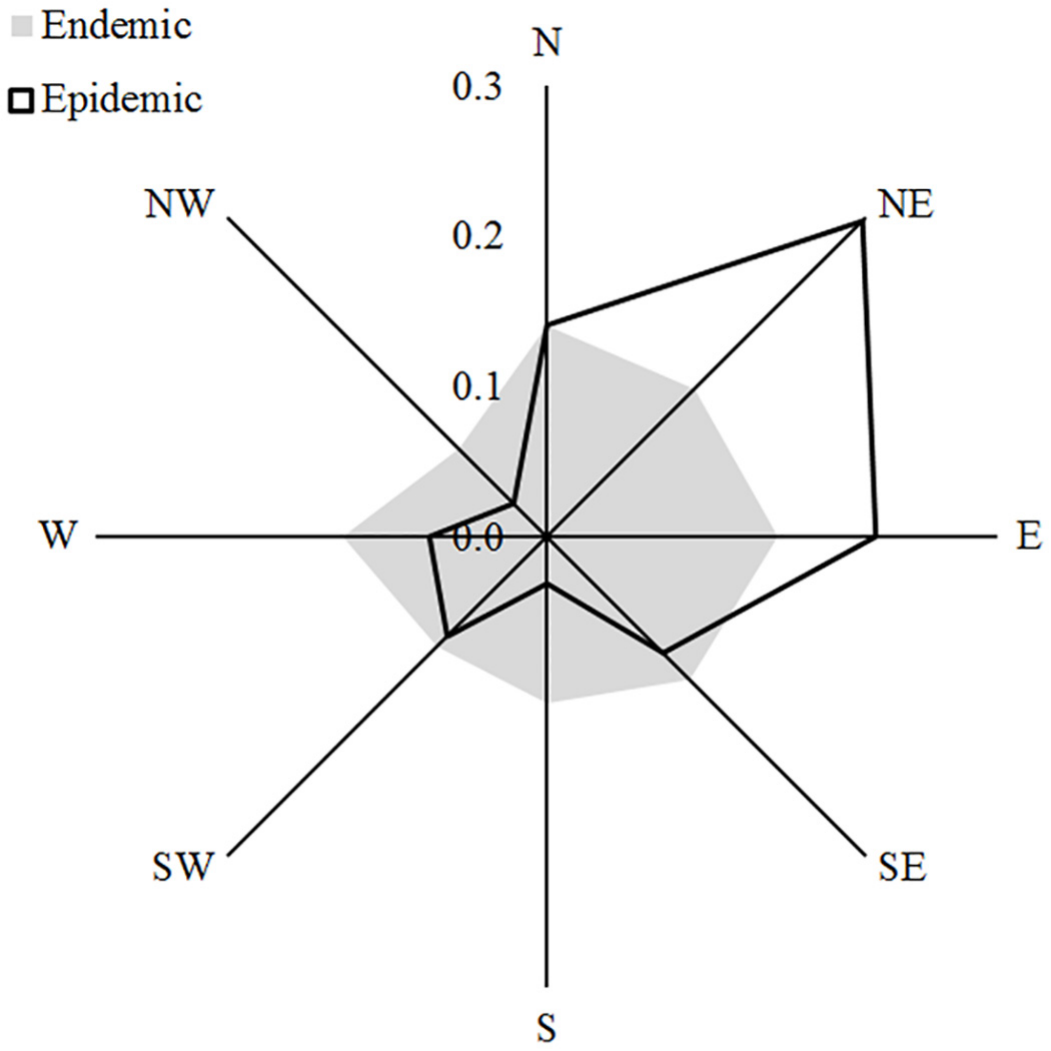
Proportion of stands with endemic and epidemic populations of *S*. *noctilio* for each aspect class.

To assess how *S*. *noctilio* outbreaks relate to environmental variables at the landscape scale, we included four environmental variables in the logistic model regressions (Tables 1 and 2). The model with the lowest AIC score contained three variables: presence of *P*. *contorta* and aspect, which were positively related with *S*. *noctilio* outbreak occurrence; and distance to nearest outbreak, which was negatively related with *S*. *noctilio* outbreak occurrence (Table 3, Fig 2).

**Table 1:** Environmental characteristics (mean ± standard error) of pine plantation stands with and without *S*. *noctilio* outbreaks in the three surveyed sites.

Variable	Abreviation	*S*. *noctilio*		CC (p)
		Endemic (N = 677)	Outbreak (N = 64)	
Presence of *P*. *ponderosa*	PpPres	0.88 ± 0.01	0.49 ± 0.07	-0.36 (p<0.000)
Presence of *P*. *contorta*	PcPres	0.16 ± 0.01	0.79 ± 0.05	0.45 (p<0.000)
Proportion *P*. *ponderosa* 1km neighborhood	Pp1kmN	0.463	0.431	-0.03 (p>0.445)
Proportion *P*. *contorta* 1km neighborhood	Pc1kmN	0.108	0.202	0.19 (p<0.000)
Altitude (ma.s.l.)	Altitude	1077.8	1084.1	-0.07 (p<0.047)
Slope (°)	Slope	12.5 ± 0.37	9.9 ± 0.88	-0.02 (p>0.585)
Aspect (cos (45°- azimuth degrees))	Aspect	1.028	1.251	0.09 (p<0.012)
Distance to nearest outbreak (m)	DNO	1634.6 ± 49	493.7 ± 107	-0.24 (p<0.000)

Column labels CC shows the results of Spearman correlations

**Table 2:** Summary of variables included in five linear regression models with best fit values (AICc).

Models	K	AIC_c_	ΔAIC_c_	R^2^
PcPres + Aspect + DNO	4	291.3	0.000	0.420
PcPres + DNO	3	293.3	1.982	0.409
PcPres + Slope + Aspect + DNO	5	293.7	2.327	0.420
PcPres + Slope + DNO	4	295.5	4.167	0.409

Variables, number of parameters in the model (K), Akaike’s Information Criterion adjusted for small sample size (AICc), difference of AICc between a model and the model with the lowest AICc (ΔAICc), and Nagelkerke’s R^2^ values, are given for each model. See Table 1 for descriptions of environmental variables studied.

**Table 3:** Parameters of the selected linear regression model (β, estándar error, and p value). See Table 1 for descriptions of environmental variables.

Parameters	β	SE	p- value
Intercept	-3.430	0.441	0.000
PcPres	2.985	0.346	0.000
Aspect	0.490	0.242	0.043
DNO	-0.001	0.000	0.000

We found that Ivlev’s electivity index, was 0.603 for *P*. *contorta* stands, and -0.621 for *P*. *ponderosa*, indicating a clear selection of *S*. *noctilio* for *P*. *contorta* stands. We also found that the proportional similarity index (PS), was PS = 0.361. This indicates a relatively narrow niche breath of *S*. *noctilio* eruptive populations in terms of host use, which means that the woodwasp only reaches outbreak levels in stands composed of referred host species.

### Spatial distribution at stand scale

At the stand scale, within the 20 sampled stands with *S*. *noctilio* presence, 72% of the sampled trees were *P*. *contorta*, 18% *P*. *ponderosa*, and 10% *P*. *radiata*. The proportion of attacked trees was significantly different among pine species (Chi^2^ = 31.22, p < 0.001). *P*. *contorta* was the species with the highest percentage of attacked individuals among the available individuals of the same species (24% of the total available trees were attacked), and was significantly more attacked than *P*. *ponderosa* (4% of the total trees where attacked, Z = 3.82, P < 0.001) and *P*. *radiata* (1% of the total trees where attacked, Z = 4.78, p < 0.001). We also found that among 280 trees sample in each of the 20 sites, the proportion of attacked trees in stands that were thinned was significant lower than in those that were not (Mann-Whitney U test, Z = 2.08, p<0.04), while no significantly differences in proportion of attacked trees were found among stands that were and were not pruned (Mann-Whitney U test, Z = 0.31, p<0.76).

**Fig 3:**
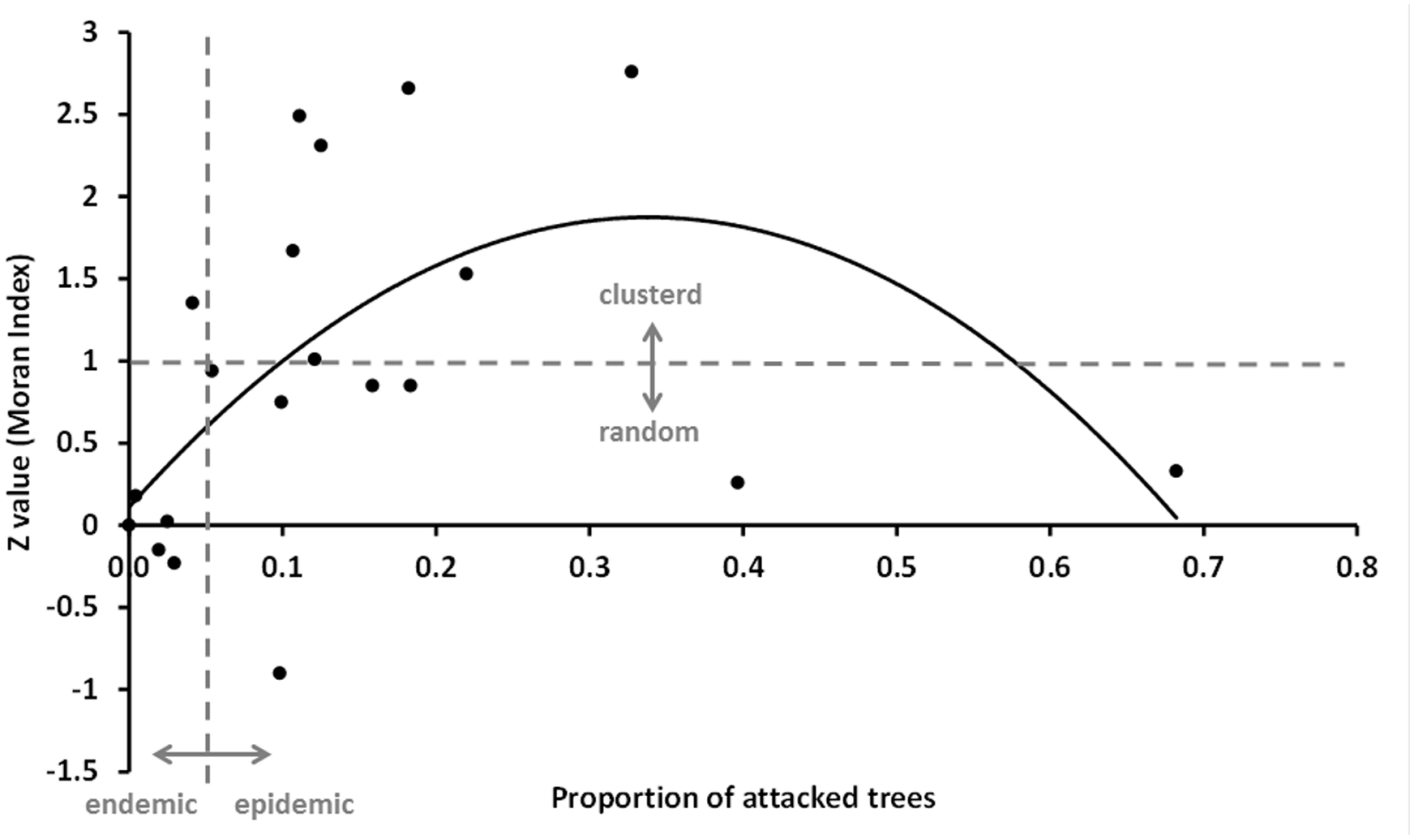
Relationship between the proportion of attacked trees by *S*. *noctilio* and the level of aggregation of the attack (Moran’s general autocorrelation index) for each of the 20 sampled stands.

We observed that the spatial arrangement of attacked trees varies according to woodwasp population density (Fig 3). We found a significant quadratic relationship between the level of aggregation of the attacked trees, and the total proportion of attacked trees in the stand: y = -15.4x^2^ + 10.4x + 0.11 (F = 3.89, p<0.041, R^2^ = 0.314). At endemic densities, the pattern of distribution of the attack is random. But, when the density of attack starts to increase towards epidemic values, the attack becomes clustered, showing the highest level of aggregation at densities of about 30% of attacked trees. At even higher attack densities, the distribution of attacked trees then becomes random again (Fig 3).

## Discussion

The spatial pattern of pine stands with *S*. *noctilio* outbreaks in NW Patagonia, is mainly influenced by the relative abundance of the host species present, the topographic aspect (i.e. north and east slope aspects), and the distance to other outbreaks, at a landscape scale. In turn, at the stand scale, we noted a clear aggregation of the attacked trees in stands with increasing infestation levels, and again, the degree of *S*. *noctilio* attacks is strongly influenced by the host species abundance and by plantation thinning. Generally our findings confirm, as it has been observed for other forest pests around the world, that scale dependent factors interplay in determining the magnitude of the impact of *S*. *noctilio* in pine plantations, either directly by influencing the underlying woodwasp population dynamics, or indirectly by determining the susceptibility of the host resources to attack [12, 39–41].

At the landscape scale, we found that stands with *P*. *contorta* were preferred significantly more by *S*. *noctilio*, than any other species planted in the region. Although *P*. *ponderosa* is much more abundant than *P*. *contorta* in all three afforested landscapes we studied here (*P*. *ponderosa* was present in 88% of the stands, while *P*. *contorta* was present in only 16% of the stands), we found that in most stands in which *S*. *noctilio* outbreaks were observed (79%), the most common tree species was *P*. *contorta*. This strong preference of *S*. *noctilio* for *P*. *contorta* is probably due to differences in physical and chemical defenses of the tree species to woodwasp attacks. *P*. *ponderosa* has been found to produce higher amounts of resins than *P*. *contorta*, and has a bark that is thicker than that of *P*. *ponderosa* which could negatively affect preferences and successful oviposition by *S*. *noctilio* females (Martinson et al. in preparation). While no *Pinus* species has shown resistance against *S*. *noctilio* attacks along the invaded regions in the world, it is well known that some species are more vulnerable than others. For example, in Uruguay and Brazil *Pinus elliottii* appears to be less affected by woodwasps than *Pinus taeda*, while in South Africa, *S*. *noctilio* appears to prefer *P*. *patula* over *P*. *elliottii* [12, 42, 43]. Within the current distribution of *S*. *noctilio* in North America, *P*. *strobus* and *P*. *resinosa* seem to be the species being more heavily attacked, although non-native *P*. *sylvestris* plantations are also suffering heavy mortality [44].

We also found a significant effect of slope aspect on the probability of occurrence of *S*. *noctilio* outbreaks. Topographic characteristics such as slope aspect are known to influence site moisture conditions, and thus may trigger tree stress [39, 45]. In NW Patagonia, slope aspect is a key topographic variable influencing site moisture availability. North-facing slopes receive more solar radiation, experience greater evapotranspiration, and have less plant available soil moisture compared to south-facing slopes. Western aspects, on the other hand, are more exposed to the dominant winds, which are associated with higher precipitation and lower frequency of frosts [46]. Consequently, for plantations carried out on slopes as is commonly observed in Patagonia, spatial and temporal variations in site moisture conditions may influence the spatial pattern and severity of drought induced tree stress. Since physiologically stressed trees are presumed to be more attractive and susceptible to *S*. *noctilio* attacks [47], we suggest that slope aspect influences the occurrence of *S*. *noctilio* outbreaks through its effects on host susceptibility. It has been shown that topographical aspect can also influence infestation levels in other forest pests such as the hemlock woolly adelgid (*Adelges tsugae*) and the Douglas-fir bark beetle (*Dendroctonus pseudotsugae*) [39, 40, 48]. In line with our findings, all these studies found more frequent insect pests attacks in warmer and more xeric slopes, and concluded that trees growing on these slopes might grow less vigorously due to increased moisture stress, becoming more susceptible to insect attacks.

Outbreak occurrence probability is higher when outbreaks are occurring in stands neighboring others with outbreaks (average of approximately 500 m). This pattern is not unexpected and suggests that stands with woodwasp epidemic population levels tend to occur near extant infestations. Although similar patterns may arise from multiple confounding processes [5], this aggregated pattern of outbreaks at landscape scales is possibly due to density-dependent dispersal from nearby pest-ridden source populations. Dispersal has been identified as an important factor determining the spread of epidemics in forest insect populations [5]. New outbreak spots developing in the vicinity of existing infestations are largely dependent on short-range movement of insects in relation to various host tree, stand, and site characteristics, as well as weather conditions [49]. *S*. *noctilio* females vary greatly in body size and in dispersal potential: the smallest individuals fly short distances, and the larger females display long and continuous flight patterns [50]. Although there is no information on whether dispersal occurs as a density dependent process in *S*. *noctilio*, still larger numbers of flying females leaving plantations are likely to be observed with increasing population density (Villacide unpublished data).

The particular scale at which insects respond to landscape characteristics will depend on the species life traits, particularly their size and mobility [51]. In this sense, *S*. *noctilio* is a highly mobile species and is thus expected to be affected by larger scales. In accordance with our results, Dodds, Garman and Ross [41] found a clustered pattern of *Dendroctonus pseudotsugae* epidemic populations, with an average nearest neighbor distance of 500 m, while Wichmann and Ravn [52] found that during population outbreaks of *Ips typographus* in Denmark, most attacks occurred within 250 m of previous attacks. These authors interpreted the observed clustered patterns of outbreaks as a result of two possible biological factors, either susceptible trees are likely spatially grouped because of local environmental factors (management, topography, pathogens, etc.); or else there are density-dependent factors that influence local insect populations, and in years of high pest numbers, more individuals would be attracted to stands undergoing attack and ultimately shift to other host trees within those stands [41].

At the stand scale, we observed spatial aggregation of attacked trees at stands with intermediate levels of attacked trees. Our findings show that at endemic population levels of the wasp (i.e.: < 5% attacked stems), attacks are randomly distributed along the stand. While, at the onset of an outbreak, attacked trees start to become aggregated at multiple simultaneous epicenters along the stand, during more advanced stages, (> 30% of attacked trees) aggregates coalesce, and the distribution of healthy and attacked trees starts to become random again. Our results agree with previous studies of the spatial distribution of attacked trees by *S*. *noctilio* at the stand scale that showed a strong spatial aggregation of *S*. *noctilio* within a pine plantation during the early stages of the outbreak [7]. These authors suggested then, that aggregation behavior of *S*. *noctilio* females could increase performance through the concentration of attacks on trees. Additionally, Aparicio, Corley and Rabinovich [28] developed a model for woodwasp population dynamics within a pine plantation, and found that eruptive dynamics of the *S*. *noctilio* may be related to aggregation of attacks, and that outbreaks may occur in the absence of density independent factors, such as droughts. Very recently Fernandez Ajo, Martínez, Villacide and Corley [53], provide evidence that fungal volatiles are attractive to foraging *S*. *noctilio* females, again suggesting that clumping of attacks on given trees is likely expected. Whether the underlying processes are behavioral aggregation or demographic clumping, aggregation of attacks has been shown to be common strategy of several eruptive forest pest species, such as some bark beetle species (i.e. *Dendroctonus ponderosae*, *D*. *pseudotsugae*, *D*. *frontalis*) in the early stages of outbreaks [39, 54–56].

After initial aggregation, spatial patterns are often self-generating as a result of feedback loops, between organisms and their abiotic and biotic environment [27, 57]. In this sense, we found that some other stand-level factors, such as the planted species and some forest management practices are also important in determining the number of attacked trees. Even during an outbreak and when the abundance of wasps is very high and in stands with more than one pine species, *S*. *noctilio* significantly prefers to attack the one species (*P*. *contorta*) over several other (*P*. *ponderosa and P*. *radiata*). We also found that pine stands that were thinned are significantly less attacked than those that are overstocked. Thinning reduces competition for resources, reducing stress, and thus tree susceptibility to pest attacks [30, 56, 58, 59].

### Integrating multiple scales: implications for pest management

Studying the spatial configuration of *S*. *noctilio* populations provides insights into how outbreaks develop. Our results suggest that the observed aggregated distribution pattern of attacked trees at different spatial scales is influenced by a combination of both *S*. *noctilio* inherent population dynamics and behavior, and the distribution and availability of susceptible trees. We found that, at a landscape scale the probability of wasp populations reaching outbreak levels in any given stand is significantly higher when next to it, is another stand bearing a recent outbreak. Also, outbreaks are more likely in *P*. *contorta* dominated stands. Not unexpectedly, the observed spatial pattern of *S*. *noctilio* outbreaks distribution at stand and landscape scale, is a combination of habitat heterogeneity and population dynamics (population growth and female dispersal).

Information on tree mortality levels resulting from pest infestations at multiple spatial scales is critical for assessing pest migration rates, the extent of tree damage and decline and in determining specific factors responsible for the observed patterns [6, 12, 39, 40]. This information is also relevant for anticipating future outbreaks and prioritizing management efforts [57, 60]. It is important to note that temporal patterns are also important to understand *S*. *noctilio* population dynamics. Here, we did not explore this or else effects of factors such as weather and natural enemies, which are likely to influence the occurrence and intensity of *S*. *noctilio* outbreaks. Further studies considering both spatial and temporal dynamics of the pest at different scales will help to better identify potential mechanisms driving *S*. *noctilio* population eruptions.

At the stand scale, some management practices, such as thinning are important to reduce host susceptibility to *S*. *noctilio* attack. In this sense, implementation of timely thinning practices, where possible, may help preventing *S*. *noctilio* outbreaks, [25, 47]. Also, as previously noted by Corley and Villacide [26], knowledge on the aggregation levels of *S*. *noctilio* attacked trees allows focalizing management actions such as monitoring and the introduction of natural enemies [26].

At the landscape scale, areas susceptible to *S*. *noctilio* eruptions could be predicted based on site attributes as the planted species, slope aspect, and distance to other outbreaks. In Patagonia, we note that special attention should be paid to stands with *P*. *contorta*. Interestingly, North America, which has natural forests of *P*. *contorta* in regions in which *S*. *noctilio* has not arrived yet, are likely to be colonized in the future [61] and should be the focus of careful monitoring. Other factors contributing to tree stress at the landscape scale, such as the slope aspect, may also be considered for anticipating future outbreaks, tracing pest risk maps and planning management in susceptible stands.

Additionally, it is essential to know the status of *S*. *noctilio* populations at the landscape scale, since it has been shown that population dynamics at the stand scale are not independent of the surrounding populations. For established populations in invaded regions, good monitoring efforts are warranted to prevent and manage potential outbreaks. Based on our results, we propose that *S*. *noctilio* outbreaks start in given specific areas that are affected by local stressing factors, and then spread out to neighboring areas through larger females flying larger distances from these epicenters. In this way, suppression of epicenter outbreaks can be expected to have a positive impact on preventing outbreaks in neighboring areas.

In conclusion, the multi-scale approach we employed in this study allowed us to identify the relative roles of fine- versus coarse-scale factors on *S*. *noctilio* eruptive dynamics. We highlight the importance considering both habitat heterogeneity and population dynamics at different spatial scales to predict and or prevent future *S*. *noctilio* outbreaks. Knowledge of these factors, how they interact, and the spatiotemporal patterns of outbreak development are critical to improve woodwasp management, and to define decisions to implement strategies intended to minimize their impacts.
